# Resident gut microbiota community determines the efficacy of soluble fiber in reducing adiposity

**DOI:** 10.3389/fmicb.2024.1392016

**Published:** 2024-04-30

**Authors:** Swang M. Shallangwa, Alexander W. Ross, Alan W. Walker, Peter J. Morgan

**Affiliations:** Rowett Institute, University of Aberdeen, Scotland, United Kingdom

**Keywords:** dietary fibers, gut microbiota, obesity, short-chain fatty acids, satiety

## Abstract

Consumption of dietary fiber has been linked to several health benefits. Among these, dietary fiber breakdown through the process of anaerobic fermentation by the colonic microbiota leads to the production of beneficial metabolites, mainly short-chain fatty acids (acetate, propionate, and butyrate), which have been implicated in reduced calorie intake. Nevertheless, the link between gut microbiota and obesity remains unclear. We investigated the effects of dietary fibers on food intake and body weight gain in two independent but similarly designed studies in rats. In the first study, the inclusion of 10% w/w pectin, fructooligosaccharides or beta-glucan (*n* = 10/group) in the diets each significantly reduced body weight gain (**‘*responders*’**) compared to the cellulose control whereas, in a closely matched, but not fully identical study (*n* = 8/group), no effect of dietary fiber on body weight (**‘*non-responders’***) was observed. The aim of this work was to explore the basis of this differential response between the two similarly designed and comparable studies, with a focus on the potential role of the gut microbiota in the control of food intake and body weight.

## 1 Introduction

The use of food constituents that naturally increase satiety and thereby decrease high-calorie intake has been proposed as a potential physiological approach to reduce obesity (Slavin, 2005; Adam et al., 2014). To this end, dietary fiber has been identified as a food component linked to decreased appetite and weight gain (Slavin and Green, 2007; Lattimer and Haub, 2010; Clark and Slavin, 2013). Dietary fibers can be insoluble molecules such as cellulose, which are poorly fermented by the gut microbiota, as well as soluble molecules (pectin, inulin, fructooligosaccharides, beta-glucan) that are favored fermentable substrates for gut microbes (El Khoury et al., 2012; Mudgil and Barak, 2013; Schroeder et al., 2013). Studies carried out with murine models have demonstrated that diets supplemented with various amounts of dietary fiber can result in decreased body weight gain and/or fat mass (Slavin and Green, 2007; Adam et al., 2015; Zhao et al., 2022), although this inhibitory effect has not been universally observed (Knapp et al., 2013; Kuo et al., 2013). The reason for this inconsistency has been attributed to variability in study design such as the type of dietary fiber used, duration of feeding, dose rate, age and phenotype of the animal (Cani et al., 2007; Huang et al., 2011; Knapp et al., 2013; Schroeder et al., 2013).

The host animal’s microbiota is another potential source of inconsistency. The role of the gut microbiota in the development of obesity and energy homeostasis has been the subject of intense research (Ravussin et al., 2012), particularly since culture-independent strategies such as high-throughput sequencing have become widely adopted for gut microbiota profiling (Thursby and Juge, 2017). Generally, the murine and human gut microbiota is dominated by two phyla, namely the Gram-positive *Firmicutes* and the Gram-negative *Bacteroidetes*, although 85% of the bacterial genera found in rodent gut microbiota are not found in the human gut microbiota (Ley et al., 2005). In rodents, it has been claimed that obesity is linked to an increased abundance of the phylum *Firmicutes* with a corresponding decrease in the abundance of the phylum *Bacteroidetes* (Ley et al., 2005). However, many other studies in mice and humans have failed to demonstrate this link between weight gain and an increase in *Firmicutes/Bacteroidetes* ratio, and multiple meta-analyses have now shown that there are no reproducible microbiota composition signatures associated with obesity in humans (Finucane et al., 2014; Walters et al., 2014; Sze and Schloss, 2016).

Nonetheless, despite a lack of consistent obesity-associated microbial community patterns in humans, there are reports that have associated individual bacterial species, such as the mucin-degrading bacterium *Akkermansia muciniphila* from the phylum *Verrucomicrobia,* with the modulation of obesity (Dao et al., 2016; Abuqwider et al., 2021). Rodent-based studies have suggested that *A. muciniphila* may act by regulating metabolism and energy homeostasis and improving the sensitivity of the cells to insulin and glucose homeostasis (Abuqwider et al., 2021). *Christensenella minuta,* from the phylum *Firmicutes*, is another human gut bacterium that has received attention based on its proposed potential to prevent adipogenesis (Mazier et al., 2021). Lastly, Ravussin et al. (2012), reported that a bacterium belonging to the genus *Allobaculum,* family *Erysipelotrichaceae,* was enriched in weight-reduced mice and correlated negatively with leptin concentration.

Further evidence suggests that certain gut microbiota activities may play a role in obesity. For example, the fermentation of dietary fiber by gut microbes results in the production of short chain fatty acids (SCFAs), mainly acetate, butyrate and propionate, which activate several G-protein coupled receptors (FFAR2 and FFAR3) along the gastrointestinal tract, thereby regulating the secretion of satiety hormones PYY and GLP-1 from enteroendocrine L-cells (Samuel et al., 2008; Tolhurst et al., 2012; den Besten et al., 2013). It has been suggested that the satiating effects observed as a result of dietary fiber consumption might be linked to the effects of these satiety hormones (Cani et al., 2004; Huang et al., 2011; Lin et al., 2013). Additionally, the SCFA acetate may have further satiety-inducing effects, purportedly by stimulating the orexigenic pro-opiomelanocortin (POMC) and inhibiting the anti-orexigenic Agouti-related peptide (AgRP) neurons in the arcuate nucleus of the hypothalamus (Frost et al., 2014).

In this study, we explored the link between different types of dietary fibers, gut microbiota composition and energy balance by analyzing two studies of similar design that were characterized by opposing outcomes. In the first study, Sprague Dawley rats were fed 4 different types of dietary fiber for 4 weeks, which resulted in decreased body weight gain and/or fat mass (***responders***) (published previously by Adam et al., 2014). By contrast, in a second study (*de novo* data) no changes in these parameters were observed (***non-responders***). This second study was designed to be similar to the first, albeit with a couple of important differences. FOS was replaced by inulin. As we had not anticipated, the lack of response in the second study this change was made as it had been noted in other studies that inulin may act via acetate to control food intake (Frost et al., 2014), which was of interest to study. Apart from this, there was close similarity in design between the two studies, and thus the discordant phenotypic responses between the two studies raised questions about whether these differences could be due to differences in the gut microbiota of the rats.

## 2 Materials and methods

### 2.1 Diets

Diets based on the AIN-93M Diet (American Society of Nutrition, Bethesda, MD, USA) were used to maintain adult rats [Special Diet Services Ltd., Witham, Essex, United Kingdom (SDS)]. For the ***responder*** study, control diets contained 5% w/w cellulose (CONT) and experimental diets contained either 10% w/w cellulose (CELL), fructooligosaccharides (FOS), beta-glucan (GLUC) or apple pectin (PECT) (Supplementary Table S1; Adam et al., 2014). All diets were isocaloric. One week before the commencement of the experiment, all rats were acclimatized on a purified low-fat diet containing 5% cellulose.

In the ***non-responder*** study, diets were also isocaloric and the control diet included 5% w/w cellulose (CELL: SDS), while the experimental diets each had 10% w/w inulin (INU), beta-glucan (GLUC) or pectin (PECT): inulin (chicory root), product code P09025; beta-glucan (oat), product code P02022; pectin (apple) product code P01253, all Cambridge Commodities Ltd. Ely, Cambridgeshire, England. Diets were prepared by SDS (Supplementary Table S2). Like the ***responder*** study, rats were acclimatized on a purified low-fat diet containing 5% cellulose 1 week prior to the commencement of the experiment.

### 2.2 Experimental animals, study design and sample collection

Animal experiments were conducted in line with UK Home Office Animal (Scientific Procedures) Act 1986, conforming to Institutional and national guidelines for the care and use of animals, and with approval by the local ethical review board (AWERB) at the University of Aberdeen. In the ***responder** study* (Adam et al., 2014), 50 young adult outbred male Sprague Dawley rats were obtained from Charles River Laboratories (Charles River Laboratories, Tranent, East Lothian, UK) and housed singly in cages in rooms maintained at 21 ± 2°C and 55 ± 10% humidity. The bedding was sawdust with shredded paper for nesting with plastic tunnels as enrichment. Water was provided *ad libitum,* and the photoperiod was standard 12 h:12 h light: dark cycle. The ***responders*** study was carried out at the Rowett Institute, when it was located in Bucksburn, Aberdeen. Subsequent analysis, including fecal microbial DNA extraction and PCR (Polymerase Chain Reaction) amplifications, were conducted at the Rowett Institute building located in Foresterhill, Aberdeen. Sequencing was carried out by the Center for Genome-Enabled Biology and Medicine (CGEBM) at the University of Aberdeen.

For the ***non-responders*** study, 32 young adult male Sprague Dawley rats were obtained from Charles River Laboratories (Charles River Laboratories, Tranent, East Lothian, UK) and housed under closely matched conditions to the ***responder*** study, although the ***non-responder*** study was carried out at the Medical Research Facility (MRF) building located at the University of Aberdeen. The ***non-responder*** study was carried out under UK Home Office project license number P5ACD03D2 with local study plan number 140317AR. As with the ***responder*** study, the fecal microbial DNA extraction and PCR amplifications were performed in the Rowett building located at Foresterhill, Aberdeen with sequencing also carried out by CGEBM at the University of Aberdeen.

In the ***responder*** study, following 7 days of acclimatization to a diet containing 5% w/w cellulose, 12-week-old rats were randomly placed in weight matched groups and offered experimental pelleted diets (10% w/w CELL, FOS, GLUC, or PECT) *ad libitum* for 28 days (*n* = 10) and a control group that continued consuming the 5% w/w cellulose diet (CONT). In ***non-responders***, after 7 days of acclimation on a 5% w/w cellulose diet, the 12-week-old rats were randomly assigned to one of 4 groups, including one control diet that contained 5% cellulose, while the rest were placed on the experimental pelleted diets (10% w/w GLUC, INUL or PECT) *ad libitum* for 28 days (*n* = 8). Random weight matching was achieved by ranking the animals from lightest to heaviest, then assigning them with random numbers using Rand in Excel (Microsoft Corporation, Redmond, WA, USA). For the ***non-responder*** study, this produced 8 groups of 4 animals. From the lightest group of four animals, the animal with the smallest random number was assigned to group 1, next smallest random number to group 2 and so on for this and each of the remaining 7 groups, to produce four treatment groups, each of 8 rats with similarly matched average weight ranges. The 4 groups were then assigned to the diets randomly by animal house staff who were unaware of the group weights, and this assignment allowed subsequent blind analysis by the research team.

Daily measurements of voluntary food intake were recorded from the weight difference of food provided and remaining each day. This involved weighing the pelleted food in the food hopper from which the animals could eat and any food spillage in the cages. The use of colored food pellets facilitated the identification of spilled food. Body weights were recorded twice a week. Magnetic resonance imaging (MRI; EchoMRI 2004, Echo Medical Systems, Houston, TX, USA) was used to determine body composition in conscious rats on day 0 and day 28 of both experiments. The MRI technique measures the fat and lean mass of tissues in live animals utilizing nuclear magnetic resonance (NMR) technology (Mystkowski et al., 2000).

In both studies, following completion of the last MRI scans, rats were killed by decapitation under inhalation anesthesia (isoflurane; IsoFlo®, Abbott Animal Health, Maidenhead, Berkshire, UK), approximately 1–3 h after lights-on. Terminal (trunk) blood was collected into chilled tubes containing EDTA as an anticoagulant and a peptidase inhibitor cocktail containing general protease inhibitor (cØmplete; Roche Diagnostics Ltd., Burgess Hill, West Sussex, UK) and a dipeptidyl peptidase-4 inhibitor (Ile-Pro-Ile; Sigma-Aldrich, Gillingham, Dorset, UK), then centrifuged immediately at 3000 × *g* for 10 min, and plasma stored at −20°C until needed for analysis. Experimental design and all animal procedures, data collection, analyses and statistics were conducted in adherence with the Arrive guidelines.^1^

### 2.3 Plasma analysis for PYY and GLP-1

In the ***responder*** study, the plasma levels of gut hormones were analyzed using commercial radioimmunoassay kits according to the manufacturer’s instructions. Kit GLP1T-36HK (Merck Millipore, Billerica, MA, USA; lower detection limit: 3 pM) was used to measure total GLP-1, while kit GLP1A-35HK (Merck Millipore; lower detection limit: 3 pM) was used to measure the biologically active form of GLP-1 namely GLP-1(7–36) amide or GLP-1 (7–37). Kit RMPYY-68HK (Merck Millipore; lower detection limit 15.6 pg/mL) was used to measure both biologically active forms of PYY, i.e., PYY (1–36) and PYY (3–36). For the ***non-responder*** study, plasma PYY was analyzed using Millipore’s MILLIPLEX® MAP Rat Metabolic Hormone Magnetic Bead panel following the manufacturer’s instructions and using a Luminex 200 instrument (Merck Life Science UK Limited, Gillingham, Dorset, UK). Plasma GLP-1 was analyzed using a total GLP-1 ELISA kit (Merck Life Science UK Limited, Gillingham, Dorset UK; cat no EZGLP1T-36K) also following the manufacturer’s instructions and using a μQuant Microplate Spectrophotometer (Biotek, Winooski, Vermont, USA).

### 2.4 Analysis of gut microbiota fermentation products

In both studies, the products of gut bacterial fermentation (i.e., short-chain fatty acid) were measured by capillary gas chromatography using the technique developed by Richardson et al. (1989) with helium used as the carrier gas. Samples were diluted for a short time in distilled water (1:4 dilution) and 2-ethylbutyric acid (5 mmol/L) was added as internal standard. The extraction of samples was then carried out in diethyl ether and derivatized with N-tert-butyldimethylsilyl-N-methyltrifluoroacetamide. Analysis was carried out on Agilent GC HP-1 capillary columns.

### 2.5 DNA extraction

Cecal contents from the ***responder study*** (Adam et al., 2014) were stored in −70°C for 6 years prior to microbial DNA extraction. Cecal contents of ***non-responder*** study were stored in −20°C with microbial DNA extracted in the same year. DNA was extracted using the FastDNA® SPIN Kit for Feces (MP Biomedicals 116570200, MP Biomedicals SARL, Illkirch, France) and was processed according to the manufacturer’s instructions in all the studies.

### 2.6 PCR amplification of 16S rRNA genes and Illumina MiSeq sequencing

The DNA extractions were used as templates to amplify the V1-V2 variable regions of bacterial 16S rRNA genes employing forward primer MiSeq-27F (5′-AATGATACGGCGACCACCGAGATCTACACTATGGTAATTCCAGMGTTYGATYMTGGCTCAG-3′) and MiSeq-338R (5′-CAAGCAGAAGACGGCATACGAGAT-barcode-AGTCAGTCAGAAGCTGCCTCCCGTAGGAGT-3′), which also contain adaptors used for downstream Illumina sequencing. A unique 12-base pair barcode for each sample amplified was also included on the reverse primers for sample identification (Fierer et al., 2008; Hamady et al., 2008).

The extracted DNA samples were PCR-amplified using the New England Biolabs Q5® High-Fidelity DNA Polymerase (Hertfordshire, UK). For each of the extracted DNA samples, four separate 25 μL PCR reaction mixtures (quadruplets) were prepared comprising of a mixture of 5X Q5 Buffer (5 μL), 10 mM dNTPs (0.5 μL), 10 μM F Primer (1.25 μL), 10 μM R Primer (1.25 μL), template DNA (1 μL, ave. 65 ng/μL), Q5 High-Fidelity DNA Polymerase (0.25 μL), and Nuclease-Free Water (15.75 μL). PCR conditions were set at 2 min at 98°C, then 20 cycles of 30 s at 98°C, 30 s at 50°C, 90 s at 72°C; then a final 5-min extension at 72°C followed by a holding temperature of 4°C. Following verification of satisfactory amplified products by agarose gel electrophoresis, the 25 μL quadruplicate for each sample was pooled into 1.5 mL sterile microcentrifuge tubes and ethanol precipitated. Quantification of the amplicons was carried out using the Qubit dsDNA HS Assay Kit (Invitrogen, CA, USA, Q32854). An equimolar master mix required for Illumina MiSeq sequencing was prepared using equal molar quantities from each PCR amplicon sample. Sequencing of the amplicons was carried out on an Illumina MiSeq machine by CGEBM at the University of Aberdeen.

### 2.7 Statistical analysis and bioinformatics

In both the ***responder*** (Adam et al., 2014) and ***non-responder*** studies, daily food intake and twice weekly body weight data were analyzed by repeated measures ANOVA General Linear Model with time, diet and their interaction as factors using Minitab version 16 (Minitab Inc., State College, PA). Cumulative food intake and MRI data, variations in body weight, fat and lean mass during the experiment and final plasma satiety hormone concentration were analyzed by one-way ANOVA (using Minitab). Tukey’s *post-hoc* test was used for pairwise comparisons in both studies.

For the microbiota data, the resulting Illumina MiSeq sequencing datasets were analyzed using the Mothur software package (Schloss et al., 2009), based primarily on the Mothur MiSeq standard operating procedure (Kozich et al., 2013). First, the forward and reverse reads from each sample were assembled into paired contigs giving rise to a total of 1,872,738 sequences. As a quality control measure, any paired contigs that were shorter than 280 base pairs or longer than 470 base pairs, that had ambiguous bases or contained homopolymeric base stretches of 8 and above were screened and removed. Unique sequences were then aligned by mapping them against the SILVA reference database to mitigate against potential sequencing errors, the Pre-cluster command, allowing 3 base differences, was run (Huse et al., 2010). To further improve the quality of the sequences, chimeric molecules that might have been formed during the amplification process were detected by UCHIME and removed (Edgar et al., 2011). Taxonomic classification of each read was carried out by using the Ribosomal Database Project (RDP) (release 10) (Wang et al., 2007) as a reference database. Remaining reads were then clustered into operational taxonomic units (OTUs) created at 97% similarity using Mothur. Subsampling was done at 5962 reads per sample to ensure consistency for comparison. Metastats software (White et al., 2009), which incorporates Fisher’s exact test to determine whether there are any OTUs (or higher taxa) that are significantly differentiated between groups, was used to compare the groups. The focus was on OTUs that had a proportional abundance of greater than or equal to 0.5% and the *p* values generated by Metastats were corrected by the Benjamini Hochberg method (Benjamini and Hochberg, 1995) to control for false discovery rate (FDR). The Pearson correlation coefficient was used to examine the relationship between the proportional abundance of OTU4 *Allobaculum fili* and OTU2 *Allobaculum fili* (92.74%) with percentage fat mass and lean mass change.

The diversity within each sample (alpha diversity) was then measured using observed richness (sobs), estimated total richness (Chao), and Good’s coverage (Finotello et al., 2018) with Mothur software. Principle coordinate analysis (PCoA) plots were created from Bray Curtis index calculator by generating a distant matrix based on the shared file. R package ggplot2 (Wickham, 2009) was used to visualize the mappings of the different groups on the PCoA plots revealing the beta diversity of the different samples.

## 3 Results

### 3.1 Food intake and body weight

**Figure 1:**
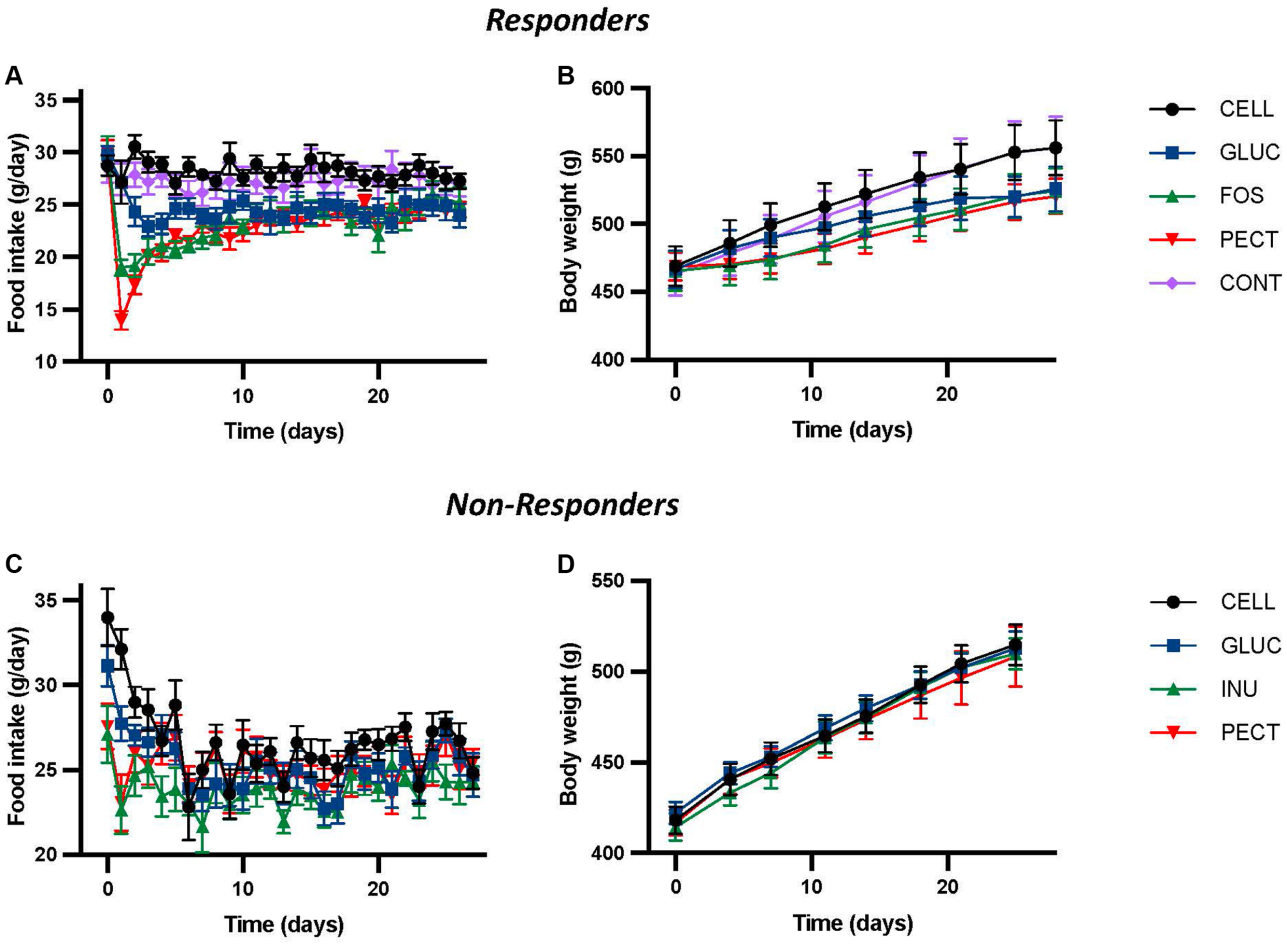
Food intake and body weight for animals in both the *responders* and *non-responders* studies. The figure shows daily voluntary food intakes **(A,C)** and body weights **(B,D)** from the ***responders*** and ***non-responders*** studies, respectively. In the ***responders*** study rats were fed 4 different dietary fibers for 4 weeks. Diets contained 10% w/w dietary fiber as cellulose (CELL, *n* = 10), 5% w/w cellulose (CONT, *n* = 10), 10% w/w fructooligosaccharides (FOS, *n* = 10), 10% w/w oat beta-glucan (GLUC, *n* = 10), or 10% w/w apple pectin (PECT, *n* = 10). In the ***non-responders*** study rats were fed 5% w/w dietary fiber as cellulose (CELL, *n* = 8), 10% w/w oat beta-glucan (GLUC, *n* = 8), 10% w/w inulin (INU, *n* = 8) or 10% w/w apple pectin (PECT, *n* = 8). Values from both studies show mean ± SEM, and data was analyzed by repeated measures ANOVA. In the ***responders*** study this revealed a significant effect of diet (*p* < 0.001), time (*p* < 0.001) and their interaction (*p* < 0.001) for food intake and a significant effect of diet (*p* < 0.003) and time (*p* < 0.001), but no interaction for body weight (*p* = 0.1). There were no statistically significant effects of diet or time in the ***non-responders*** study. Data for the ***responders*** study was redrawn from Adam et al. (2014), under CC BY 3.0 license.

In the ***responder*** study, repeated-measures ANOVA of daily food intake revealed a highly significant effect of diet, time, and their interactions (all *p* < 0.001; Figure 1A), where rats fed the soluble dietary fibers GLUC (10% w/w oat beta-glucan), FOS (10% w/w fructooligosaccharides), and PECT (10% w/w pectin) each had significantly lower daily food intakes relative to those fed either 5% (CONT) or 10% (CELL) w/w cellulose control diets (Figure 1A). There were no differences in food intake between the CONT and CELL groups, or within the soluble dietary fiber groups. Repeated-measures ANOVA of body weight also showed a significant effect of time (*p* < 0.001) and diet (*p* < 0.01), with the body weights of the GLUC, FOS and PECT groups significantly reduced relative to the CELL and CONT controls (Figure 1B; Adam et al., 2014).

In the ***non-responder*** study, no significant effects of diet, time or their interactions were found by repeated measures ANOVA (Figure 1C). While there was a significant difference in food intake (both *p* < 0.05) between control rats fed 5% cellulose (CELL) and those fed either of the fiber diets 10% w/w inulin (INU) or 10% w/w pectin (PECT) recorded in the first 3 days, this was transitory and no significant difference in cumulative food intake was observed between the groups across the 28 days of the study. Similarly, repeated measures ANOVA of bodyweight showed no significant difference due to diet, time, or their interaction (Figure 1D).

The initial percentages of total fat and lean mass at the start of the experiment for the ***responders*** were similar across all treatment groups (Supplementary Table S3; Adam et al., 2014). While there was no treatment effect on the final percentage lean mass across the experimental period, rats in the soluble dietary fiber groups [GLUC (*p* < 0.01), FOS (*p* < 0.01) and PECT (*p* < 0.001)] each had significantly reduced final percentage body fat relative to both CONT and CELL groups. Correspondingly final body weights were significantly lower in rats fed each of the soluble dietary fibers relative to both CONT and CELL controls (Supplementary Table S3). There were no significant differences in the final body weight between the rats in the experimental groups compared to rats in both CONT and CELL groups (Supplementary Table S3).

**Figure 2:**
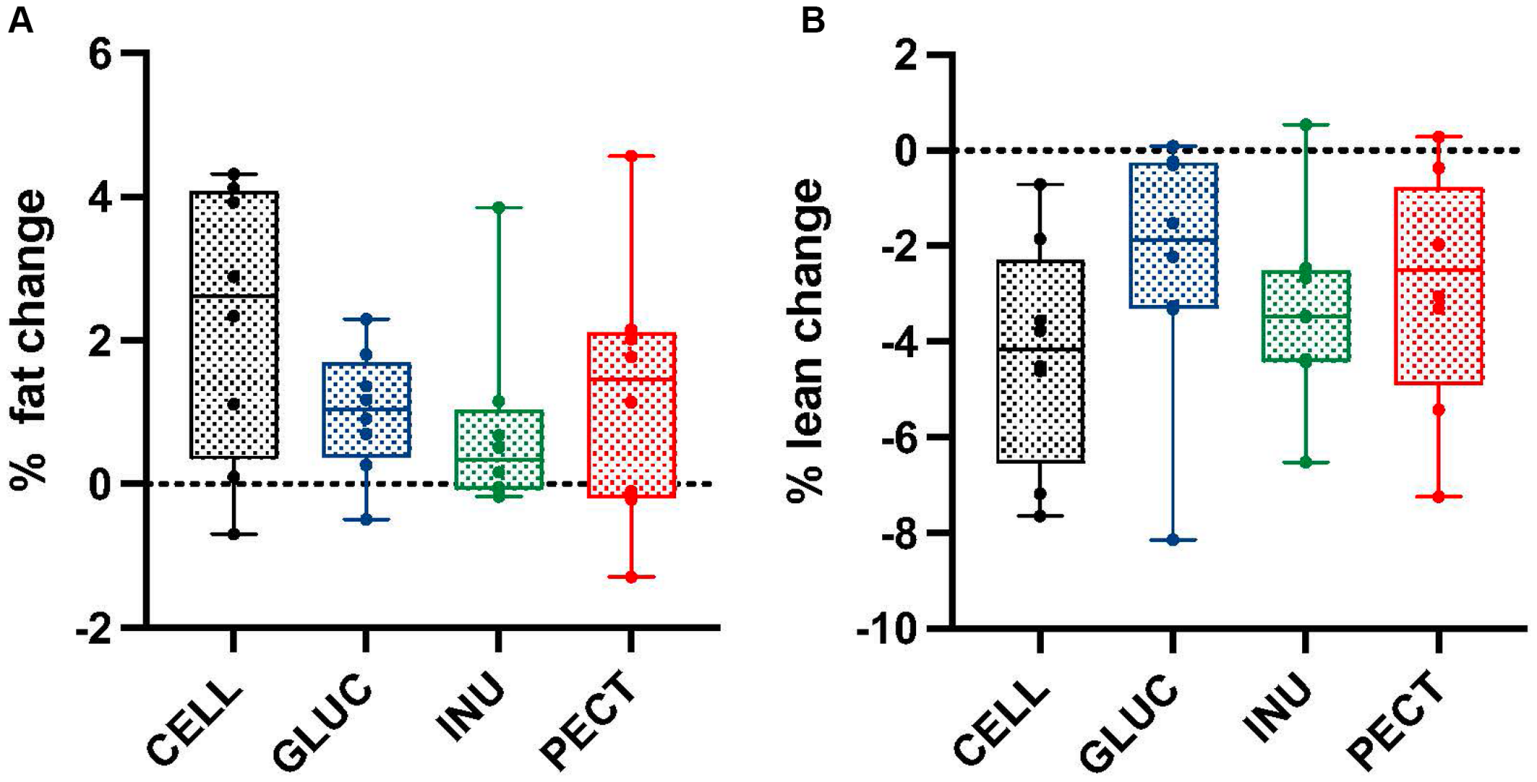
Percentage of fat and lean mass changes in samples from the *non-responders* study. Box and whisker plots showing **(A)** percentage fat change and **(B)** percentage lean change in ***non-responders***. Body composition was measured by MRI at the start (0 weeks) and end (4 weeks). Diets contained 5% w/w dietary fiber as cellulose (CELL, *n* = 8), 10% w/w oat beta-glucan (GLUC, *n* = 8), 10% w/w inulin (INUL, *n* = 8) or 10% w/w apple pectin (PECT, *n* = 8). The horizontal lines in the boxes represent the median. Data were analyzed by one-way ANOVA with Tukey’s *post hoc* test. There were no statistically significant differences between treatments.

Unlike the ***responders***, there were no significant differences or changes in percentage of fat or lean mass across all treatment groups in the ***non-responders*** study (Figures 2A,B).

### 3.2 Concentration and amount of gut microbiota fermentation acids

In the ***responders*** acetate was significantly lower in the ceca of rats in the FOS and GLU groups compared to the CELL group (Table 1). There were no significant differences in the concentration of acetate between the PECT and the CELL groups (Table 1). Propionate was significantly higher in the GLU group compared to the other groups. Butyrate was significantly higher in the FOS group compared to the PECT group (Table 1).

**Table 1:** Concentration (mmol/l) of fermentation products in the cecal samples collected as part of the *responders* study.

Cecum responders	Diet group				
	CONT	CELL	FOS	GLUC	PECT
*Concentrations (mmol/l)*					
Acetate	47.6 ± 2.72^a^	41.2 ± 3.14^a^	21.7 ± 3.51^b^	23.8 ± 3.38^b^	49.2 ± 6.00^a^
Propionate	8.6 ± 0.58^a,b^	6.7 ± 0.48^b,c^	4.0 ± 0.89^c^	11.9 ± 1.88^a^	7.8 ± 0.70^b,c^
Butyrate	7.1 ± 0.58^a,b^	8.0 ± 1.37^a,b^	10.3 ± 2.29^a^	7.5 ± 1.48^a,b^	3.9 ± 0.84^b^

Rats were given different dietary treatments. Re-used from Adam et al. (2014), under CC BY 3.0 license. Diets were control (CONT) or contained 10% w/w fiber as cellulose (CELL), fructo-oligosaccharide (FOS), oat beta-glucan (GLUC) or apple pectin (PECT). Values are means ± s.e.m. (*n* = 10/group). Within rows, values without a common letter were significantly different (*p* < 0.05).

In the ***non-responders*** acetate was significantly lower in the INU compared to the GLUC and PECT groups (Table 2). Propionate was significantly lower in the INU compared to control CELL group (*p* < 0.05). The concentration of butyrate was significantly higher in the ceca of rats belonging to the GLUC group compared to those of the INU and PECT group (Table 2).

**Table 2:** Concentration (mmol/l) of fermentation products in the rat cecal samples collected as part of the ***non-responder*** study.

Cecum non-responders	Diet group			
	CELL	GLUC	INU	PECT
*Concentrations (mmol/l)*				
Acetate	46.50 ± 2.67^ab^	63.26 ± 7.86^a^	37.11 ± 8.49^b^	65.14 ± 4.63^a^
Propionate	9.68 ± 0.91^a^	7.78 ± 1.33^ab^	5.51 ± 1.85^b^	5.86 ± 0.63^ab^
Butyrate	6.78 ± 0.50^ab^	9.27 ± 3.05^a^	1.36 ± 0.56^b^	2.61 ± 0.41^b^

Diets were control 5% cellulose (CELL) or contained 10% w/w fiber oat beta-glucan (GLUC), inulin (INU), apple pectin (PECT). Values are means ± s.e.m. (*n* = 8/group). Within rows, values without a common letter were significantly different (*p* < 0.05).

### 3.3 Satiety hormone PYY and GLP-1 concentrations in plasma

**Figure 3:**
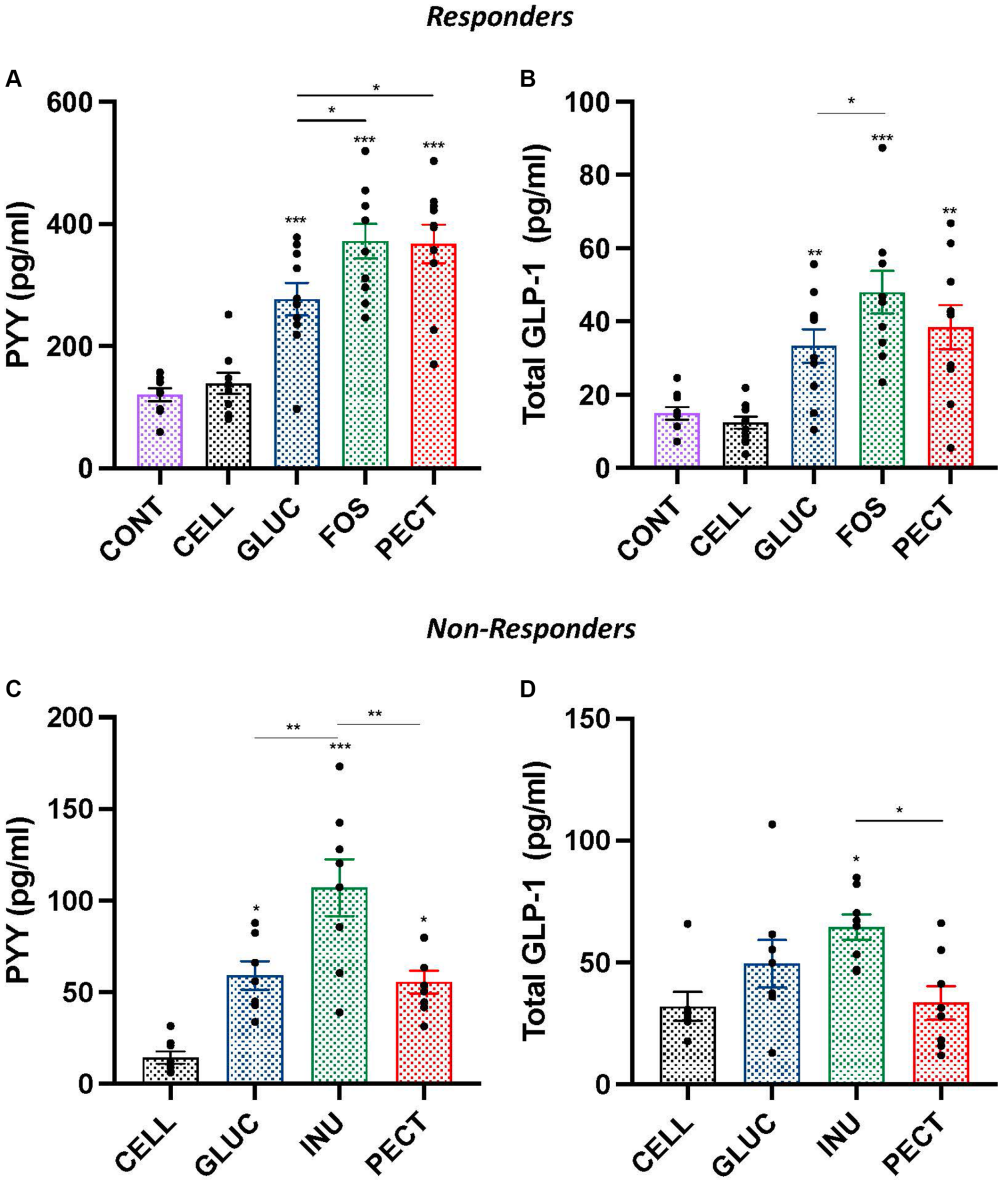
Plasma hormones concentration of gut peptide satiety hormones PYY and GLP-1 in samples from both the *responders* and *non-responders* studies. Bar charts show plasma concentration of PYY **(A,C)** and plasma concentrations of total GLP-1 **(B,D)** from *responders* and *non-responders* studies, respectively. Data were analyzed by ANOVA with Tukey’s *post hoc* test. Asterisks above individual treatment bars indicate statistical difference relative to both CONT and CELL (responders) and CELL (non-responders). Other asterisks show specific comparisons indicated by the horizontal lines, *p < 0.05, **p < 0.01, ***p < 0.001. Data for *responders* study was redrawn from Adam et al. (2014), under CC BY 3.0 license.

In the ***responders***, the plasma concentrations of satiety hormones PYY and GLP-1 were each significantly higher in the GLUC, FOS, and PECT groups compared with both the CONT and CELL groups (*p* < 0.001; Figures 3A,B). In the soluble dietary fiber groups, rats in the GLUC group had a significantly lower PYY compared to the FOS and the PECT groups (both *p* < 0.05). Rats in the GLUC group also had a significantly lower GLP-1 compared to rats in the FOS group. For PYY and GLP-1, there was no significant difference in the concentration for each hormone between the CONT and CELL groups.

The plasma concentration of satiety hormone PYY in ***non-responders*** was significantly higher in rats from the INU (*p* < 0.001), GLUC (*p* < 0.05), and PECT (*p* < 0.05) groups than in rats from the CELL group (Figure 3C). Rats in the INU group had a significantly higher concentration of PYY than rats in the GLUC and PECT (both *p* < 0.01). There was no difference in the plasma concentration of PYY between rats in the GLUC and rats in the PECT groups. The concentration of total plasma GLP-1 was only significantly higher than the CELL control group in rats belonging to the INU group (*p* < 0.05; Figure 3D). The plasma GLP-1 levels in the INU group were also significantly higher than the PECT group (*p* < 0.022). There was no significant difference in the concentration of GLP-1 between other groups.

### 3.4 Illumina MiSeq sequencing of cecal microbiota

Given that the microbiota might also have played a role in weight gain or loss, the cecal microbiota of rats in the two studies was analyzed by 16S rRNA gene sequencing with subsequent analysis using Mothur software. For the ***responders*** the 10% cellulose (CELL) group was used as the control against which the fiber treatments, oat beta-glucan (GLUC), fructooligosaccharides (FOS), and pectin (PECT) were compared. In the ***non-responders**,* the 5% cellulose (CELL) was used as the control while the 10% beta-glucan (GLUC), inulin (INU), and pectin (PECT) served as the experimental groups.

**Figure 4:**
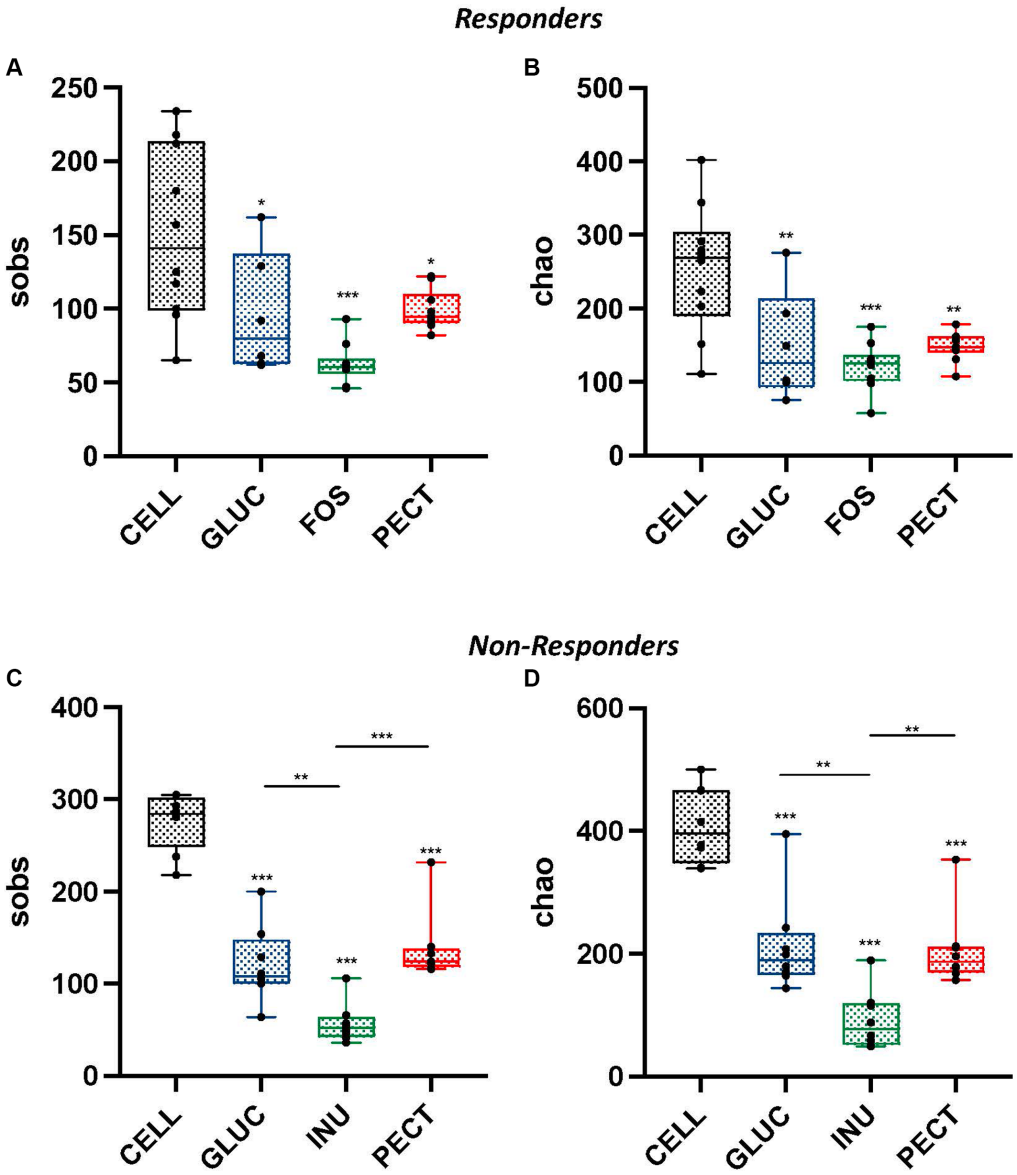
Alpha diversity measurements for cecal samples obtained from both the *responders* and *non-responders* study samples. Box and whisker plots showing observed species richness (sobs) **(A,C)** and chao estimated species richness **(B,D)** for ***responders*** and ***non-responders***, respectively. For **responders**, diets contained 10% w/w fiber as cellulose (CELL, *n* = 10), oat beta-glucan (GLUC, *n* = 6), fructooligosaccharide (FOS, *n* = 10) or apple pectin (PECT, *n* = 10) whereas **non-responder** diets contained 5% w/w dietary fiber as cellulose (CELL, *n* = 8), 10% w/w oat beta-glucan (GLUC, *n* = 8), 10% w/w inulin (INUL, *n* = 8) or 10% w/w apple pectin (PECT, *n* = 8). The horizontal line in the box represents the median. Data were analyzed by one-way ANOVA with Tukey’s *post hoc* test. Asterisks above individual treatments indicate statistical difference relative to CELL control. Other asterisks show specific comparisons indicated by the horizontal lines, *p < 0.05, **p < 0.01, ***p < 0.001.

Illumina MiSeq sequencing revealed a strong correlation between dietary fiber and composition of the gut microbiota. Comparison of alpha diversity (sobs and chao) showed that the cecal microbiota of rats in the control groups (CELL *responders* and CELL *non-responders*) had higher alpha diversities than the cecal microbiota of rats in their respective experimental groups. There were no significant differences in observed richness (sobs) and estimated richness (chao) between the cecal microbiota of rats in the soluble dietary fiber groups in the ***responders*** (Figures 4A,B).

In the ***non-responders**,* the cecal microbiota of rats in the PECT group showed higher observed species richness than rats in the INU group (*p* < 0.001; Figure 4C). Observed species richness was also significantly higher in the cecal microbiota of rats in the GLUC group compared to the cecal microbiota of rats in the INU group (*p* < 0.01). Also, in ***non-responders**,* Chao estimated species richness was significantly higher in the cecal microbiota of rats in the PECT group compared to rats in the INU group (*p* < 0.01; Figure 4D). The cecal microbiota of rats in the GLUC group also had a significantly higher Chao estimated richness than rats in the INU group (*p* < 0.01).

**Figure 5:**
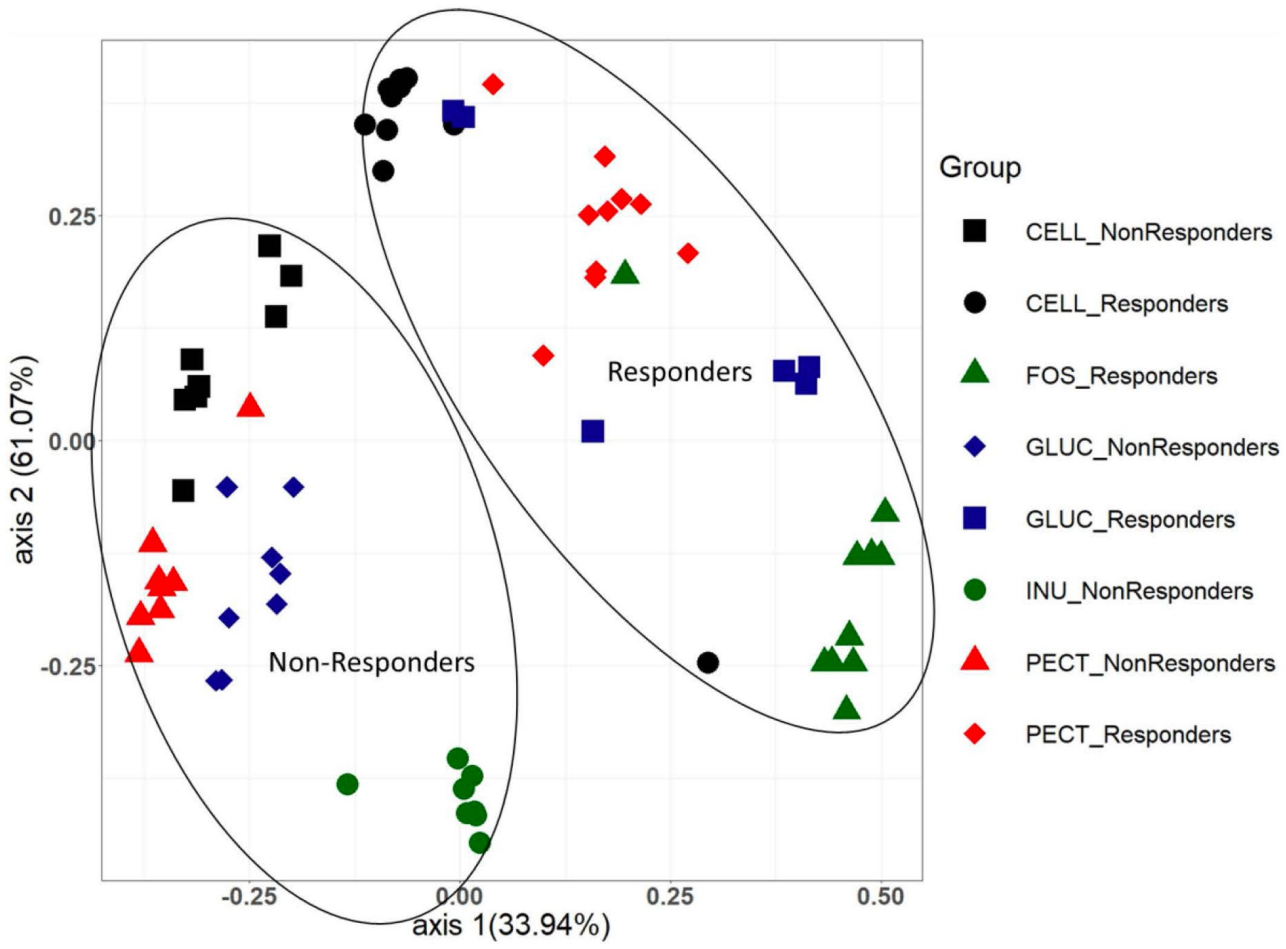
PCoA plot showing Bray Curtis beta diversity clustering patterns between cecal microbiota samples from both the *responders* and *non-responders* studies. The clustering patterns between the two different studies were confirmed as being statistically significantly different using the AMOVA (analysis of molecular variance) function implemented in Mothur.

As alpha diversity analysis indicated a significant difference between the cecal microbiota of rats in the cellulose control group compared to the rats in the soluble dietary fiber groups, further diversity analysis between the groups (beta diversity) was conducted and visualized using Bray Curtis-based principal coordinate plots (Figure 5). Nonparametric analysis of molecular variance (AMOVA) revealed a significant difference in the clustering of ***responders*** and ***non-responders***. In both studies, the cecal microbiota of rats in the control groups (cellulose) and those in the experimental groups clustered significantly differently (Figure 5). Likewise in both studies, the cecal microbiota of rats in the experimental groups clustered significantly different from each other (Figure 5).

**Figure 6:**
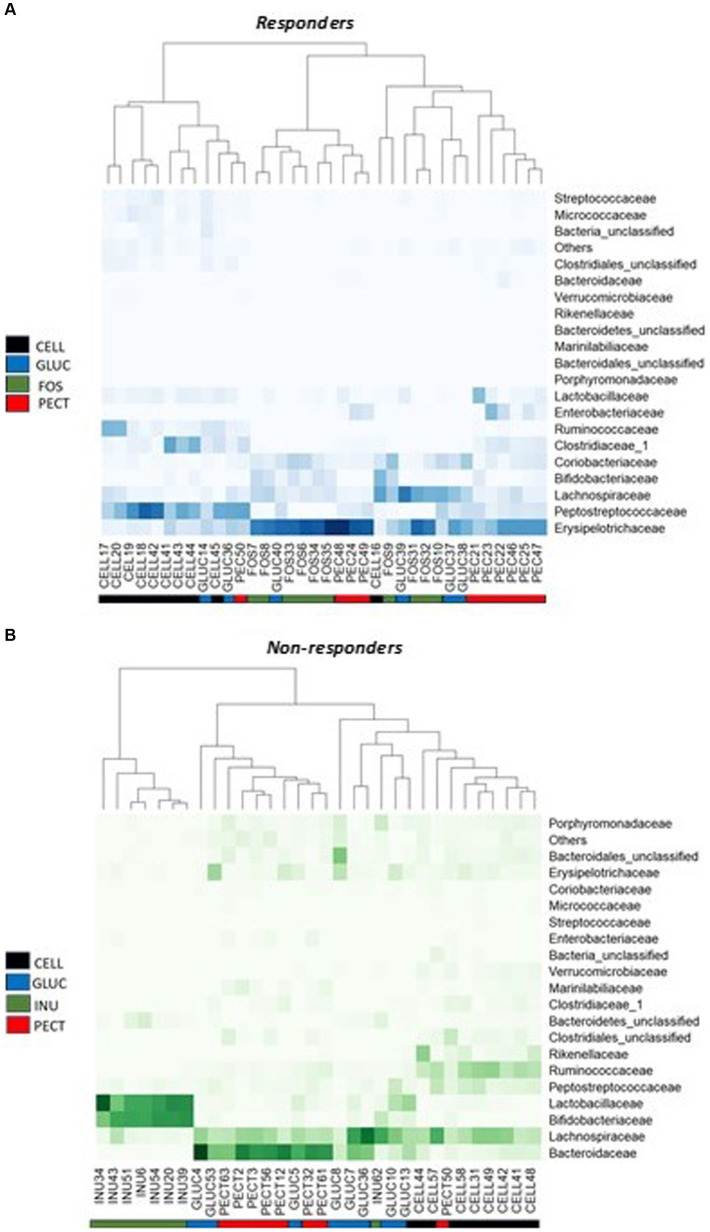
Heat map of bacterial proportional abundance at the Family level in the *responders* and *non-responders* studies. **(A)** Diets contained 10% w/w fiber as cellulose (CELL, *n* = 10), oat beta-glucan (GLUC, *n* = 6), fructooligosaccharides (FOS, *n* = 10) or apple pectin (PECT, *n* = 10). **(B)** Diets contained 5% w/w fiber as cellulose (CELL, *n* = 8), oat beta-glucan (GLUC, *n* = 8), inulin (INU, *n* = 8) or apple pectin (PECT, *n* = 8). Color intensities represent proportional abundance at the family level of classification with darker colors representing higher proportional abundance and lighter colors representing lower proportional abundance. Colored ribbons represent the different dietary fiber groups.

Having established that overall microbiota compositions differed between studies and diets, we tested whether specific bacterial taxa were associated with diets. Analysis of proportional abundance at the phylum level in the ***responders*** showed that the phylum *Actinobacteria* was significantly higher in the caeca of rats belonging to the FOS group compared to rats in control CELL (*p* < 0.05), GLUC (*p* < 0.05) and PECT group (*p* < 0.01). Also in the ***responders***, the phylum *Proteobacteria* was significantly higher in the PECT group compared to the FOS, GLUC and CELL groups (*p* < 0.01). In the ***non-responders***, *Bacteroidetes* was significantly higher in the rats belonging to the PECT group compared to rats in the INUL group and the CELL group (*p* < 0.01) while Firmicutes was significantly higher in the CELL group compared to the PECT group and the INUL group (*p* < 0.01). At the Family level of classification in the ***responders***, the percentage proportional abundance of the family *Erysipelotrichaceae* was significantly higher in the cecal microbiota of rats in the soluble dietary fiber groups FOS (50.42 ± 5.50, *p* < 0.001), PECT (44.17 ± 5.34, *p* < 0.001) and GLUC (34.67 ± 5.38, *p* < 0.05) compared with rats in the control CELL group (9.30 ± 1.76) (Figure 6A). The proportional abundance of *Erysipelotrichaceae* was not significantly different between the soluble dietary fibers. The Family *Enterobacteriaceae* in the ***responders*** was significantly proportionally higher in the PECT (9.50 ± 3.11) group compared to the FOS (0.02 ± 0.01, *p* < 0.05) and CELL (0.04 ± 0.01, *p* < 0.05; Figure 6A). In the ***non-responders***, the percentage proportional abundance of the family *Bacteroidaceae* was significantly higher in the cecal microbiota of rats from the PECT (39.41 ± 5.07, *p* < 0.01) and the GLUC groups (30.12 ± 5.73, *p* < 0.01) relative to those of the CELL control (6.63 ± 1.27) and the INU groups (0.95 ± 0.77) (Figure 6B). The percentage proportional abundance of the family *Bifidobacteriaceae* in ***non-responders*** was significantly higher in the cecal microbiota of rats in the INU group (37.84 ± 3.48) compared to the cecal microbiota of rats in GLUC (3.38 ± 1.01), PECT (0.61 ± 0.47), and CELL (0.12 ± 0.06) (*p* < 0.01; Figure 6B). The percentage proportional abundance of the family *Lactobacillaceae* was also significantly higher in the cecal microbiota of rats in the INU group (38.55 ± 5.51) compared to rats in the GLUC (8.41 ± 83.19), PECT (1.86 ± 0.46), and control CELL (1.41 ± 0.41) in the ***non-responders*** (*p* < 0.01; Figure 6B) (see also Supplementary Table S4).

**Figure 7:**
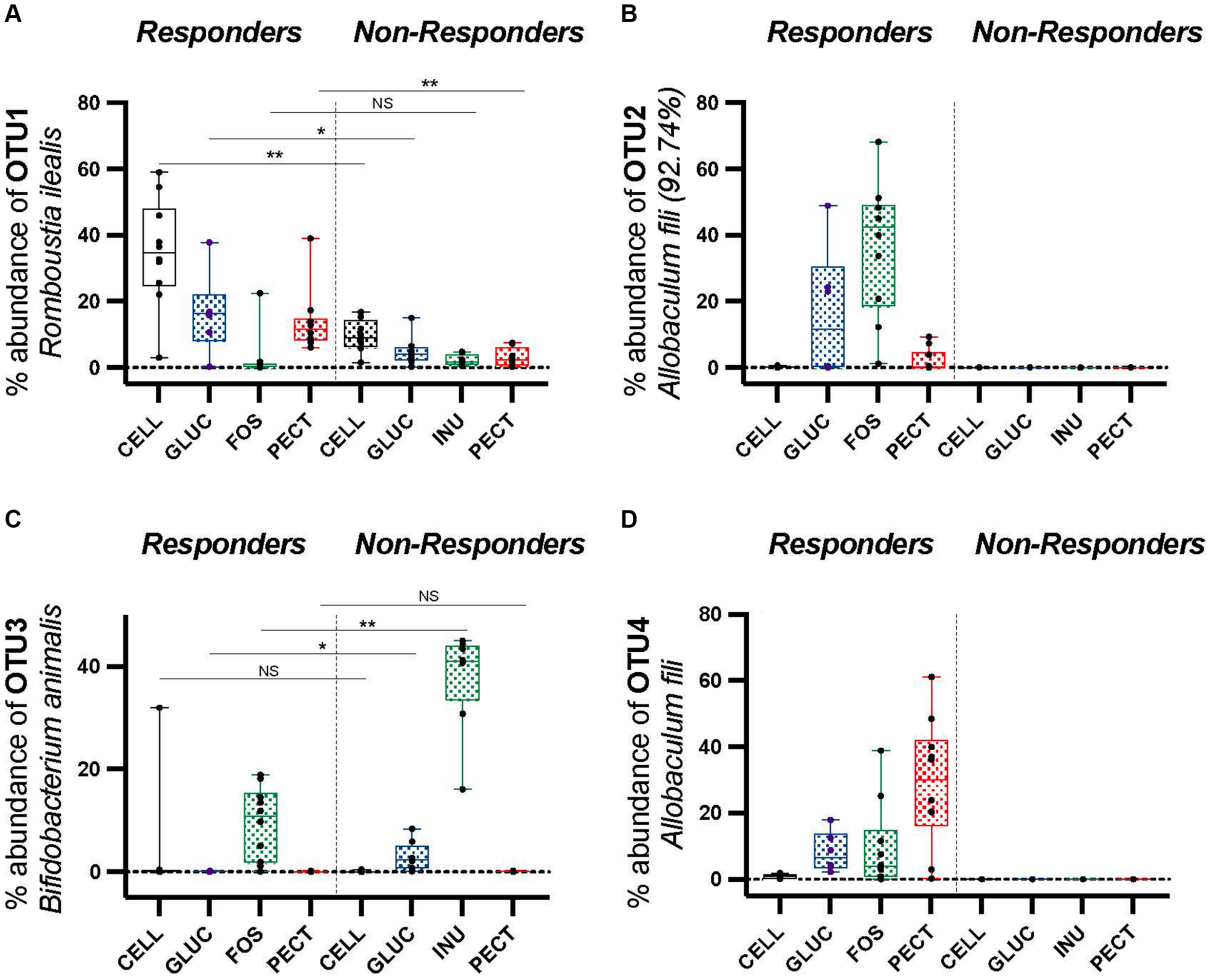
Percentage proportionally abundance of OTUs 1 to 4 across *responders* and *non-responders* studies. **(A)** OTU1 *Romboutsia ilealis* **(B)** OTU2 *Allobaculum fili* (92.74% similarity) **(C)** OTU3 *Bifidobacterium animalis* **(D)** OTU4 *Allobaculum fili*. Data were analyzed by one-way ANOVA. The horizontal lines in the boxes represent the median. Statistical significance was determined by using Metastats in Mothur and the Benjamini-Hochberg correction, except for OTU2 *Allobaculum fili* (92.74%) and OTU4 *Allobaculum fili,* as these were not detected in the ***non-responders*** (zero) so statistical tests were not applied. *p < 0.05, **p < 0.01.

Comparison of the proportional abundance of the top four most abundant OTUs in the cecal microbiota between rats from the ***responders*** and ***non-responders*** revealed that OTU1, identified as *Romboutsia ilealis*, was significantly higher in the cecal microbiota of rats in the control CELL group of the ***responders*** (34.93 ± 5.15) compared to the control CELL of the ***non-responders*** (9.62 ± 1.76) (*p* < 0.01; Figure 7A; *p* < 0.01). It was also significantly more dominant in the PECT group of the ***responders*** (13.72 ± 3.0) compared to the PECT group of the ***non-responders*** (3.18 ± 0.99) (*p* < 0.01; Figure 7A). OTU2, identified as *Allobaculum fili* (92.74% similarity), was proportionately highly abundant in the FOS group of ***responders*** (36.57 ± 6.34) but undetectable in the INU group of the ***non-responders*** (Figure 7B). The cecal microbiota of rats in the control CELL group of the ***responders*** also showed a high proportional abundance of OTU2 (0.17 ± 0.06) yet it was absent or undetectable in the control CELL group of the ***non-responder*** rats (Figure 7B). Within the ***responder*** study, OTU2 (*Allobaculum fili* 92.74%) was significantly higher in the FOS group (36.57 ± 6.34) compared to GLUC (16.14 ± 8.06), PECT (13.82 ± 2.99) (*p* < 0.05). It was also significantly higher in the FOS group (36.57 ± 6.34) compared to the control CELL groups (0.17 ± 0.06) (*p* < 0.01). Rats in the FOS group of ***responders*** harbored a significantly lower proportional abundance of OTU3 (*Bifidobacterium animalis*) (9.51 ± 2.24) compared to rats in the INU group of the ***non-responders*** (37.82 ± 3.48) (*p* < 0.01; Figure 7C). Rats in the GLUC of ***responders*** also had a lower proportional abundance of OTU3 (*Bifidobacterium animalis*) (0.67 ± 0.03) than their counterparts in the GLUC of the ***non-responders*** (29.30 ± 0.01) (*p* < 0.05; Figure 7C). Comparison of the cecal microbiota between rats on the same type of fiber between ***responders*** and ***non-responders*** showed that OTU4, identified as *Allobaculum fili*, represented a high proportion of bacteria in ***responders*** but was below the level of detection in ***non-responders*** (Figure 7D). In the ***responders**,* OTU4 (*Allobaculum fili*) was significantly higher in the PECT (29.05 ± 6.08, *p* < 0.01), FOS (9.53 ± 4.05, *p* < 0.01), and GLU (8.22 ± 2.48, *p* < 0.05), compared to the rats belonging to the control CELL group (0.8957 ± 0.20) (Figure 7D). Amongst the caecal microbiota of rats in the soluble dietary fiber groups in the ***responders**,* OTU4 (*Allobaculum fili*) was significantly higher in the PECT group (29.05 ± 6.08) compared to the FOS (9.53 ± 4.05, *p* < 0.05), and GLUC (8.22 ± 2.48, *p* < 0.05) groups (Figure 7D).

In the ***responders***, OTU4 (*Allobaculum fili*) and OTU2 [*Allobaculum fili* (92.74% similarity)] from the family *Erysipelotrichaceae* were significantly more proportionally abundant in the experimental groups compared to the control cellulose group. OTU4 (*Allobaculum fili*) was identified as significantly more dominant in the PECT (29.05 ± 6.08, *p* < 0.01), FOS (9.53 ± 4.05, *p* < 0.01), and GLUC (8.22 ± 2.48, *p* < 0.05) groups compared to the control CELL (0.8957 ± 0.20) (Figure 7D). Amongst the cecal microbiota of rats in the soluble dietary fiber groups in the ***responders***, it was significantly more abundant proportionally in the PECT group (29.05 ± 6.08) compared to the GLUC (8.22 ± 2.48) (*p* < 0.05) and FOS (9.53 ± 4.05) (*p* < 0.05; Figure 7D). Also in the ***responders***, OTU2 (*Allobaculum fili* 92.74%) was significantly more dominant in the FOS group (36.57 ± 6.34) compared to the CELL (0.17 ± 0.06) (*p* < 0.01), PECT (13.82 ± 2.99) (*p* < 0.01) and GLUC (16.14 ± 8.06) (*p* < 0.05; Figure 7B).

### 3.5 Correlation of percentage fat mass change and lean mass change with OTU4 *Allobaculum fili* and OTU2 *Allobaculum fili* (92.74%) in responders

**Figure 8:**
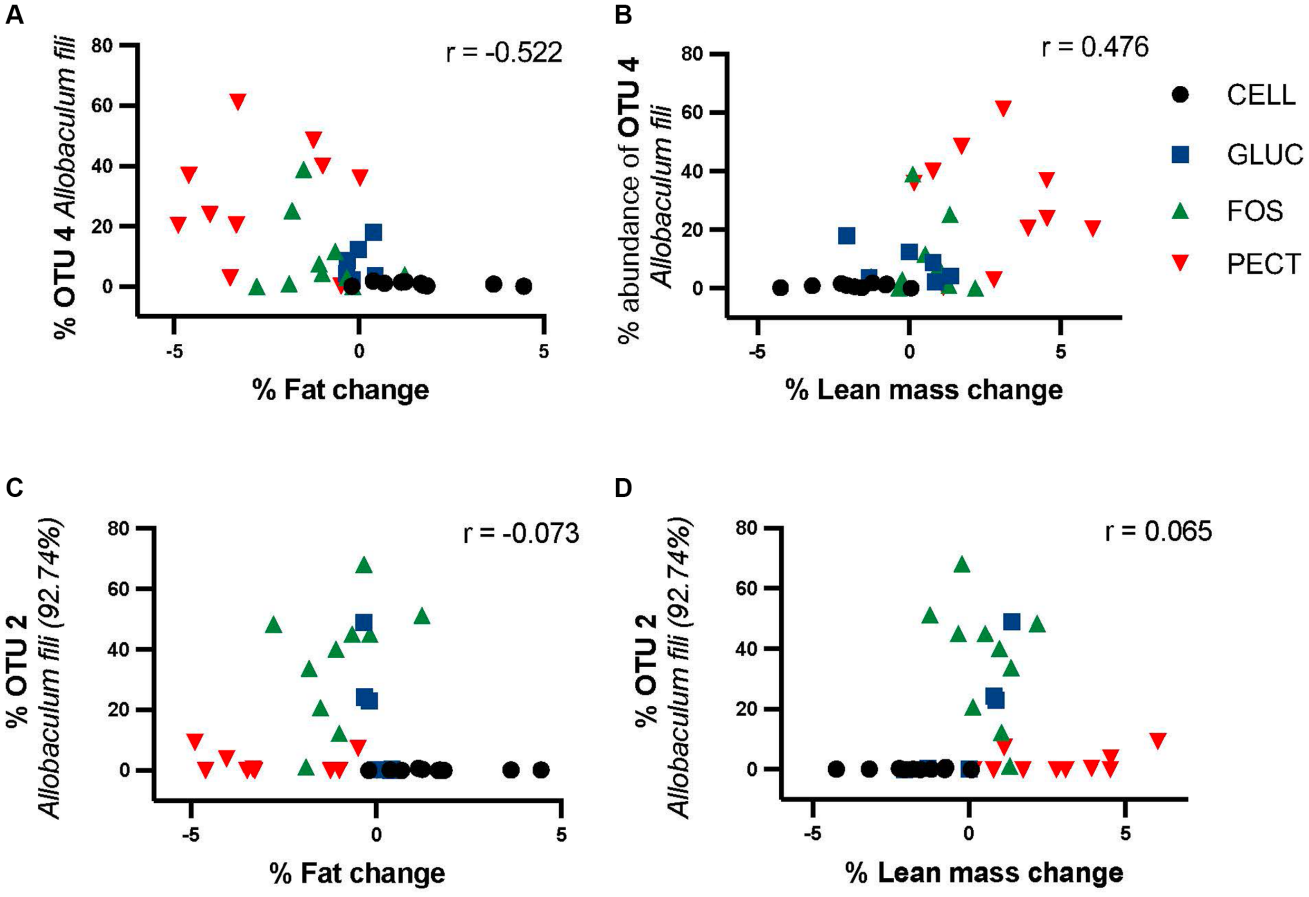
Correlation of OTU4 and OTU2 with percentage fat mass and percentage lean mass change in *responders* study samples. Percentage proportional abundance of OTU4 *Allobaculum fili* and OTU2 *Allobaculum fili* (92.74%) relative to percentage change in fat mass relative **(A,C)** percentage change in lean mass **(B,D)**. Correlation analysis (Pearson correlation coefficient) shows a significant negative correlation between the abundance of OTU4 and fat mass (*r* = −0.522) and a significant positive correlation with lean mass (*r* = 0.476). There was no significant relationship between either fat or lean mass for OTU2.

It was clear that different diets were associated with various changes in gut microbiota composition. Percentage fat mass change correlated negatively with OTU4 (*Allobaculum fili*) in ***responders*** (*r* = −0.522, *p* = 0.00153) (Figure 8A). Loss in fat mass was significantly higher in the soluble dietary fiber-fed rats than with the insoluble fiber cellulose (PECT>FOS > GLUC>CELL). Percentage lean mass change correlated positively with OTU4 (*Allobaculum fili*) (*r* = 0.47638, *p* = 0.00331) (Figure 8B). By contrast, the was no significant correlation between OTU2 and percentage fat mass (*r* = −0.073) change or OTU2 and percentage lean mass change (*r* = 0.065) (Figures 8C,D).

## 4 Discussion

Results from the ***responders*** study showed that the soluble dietary fibers pectin, fructooligosaccharides and oat beta-glucan were each associated with reduced cumulative food intake and decreased body weight gain and fat mass. This is consistent with other studies conducted in rodents, showing that consumption of soluble dietary fibers is associated with a reduction in food intake and decreased body weight gain (Cani et al., 2007; Pirman et al., 2007; Huang et al., 2011). In contrast, the ***non-responders*** study, which was designed to closely match the ***responders*** study as far as possible, failed to reproduce a reduction in food intake or decrease in body weight gain and/or fat mass associated with soluble dietary fiber intake. A similar lack of response has also been reported in other studies where rats or mice were fed different soluble dietary fibers (Knapp et al., 2013; Kuo et al., 2013; Schroeder et al., 2013). These findings lead to the general conclusion that soluble dietary fibers can reduce food intake and decrease body weight gain and/or fat mass, yet there are factors that can influence the efficacy of this dietary supplementation approach. Understanding the reasons for this inconsistency requires a better understanding of mechanisms by which dietary fiber influences the appetite regulatory system.

One plausible mechanism involves the SCFAs, which are major fermentation products of dietary fiber digestion by the gut microbiota. These are known to stimulate free fatty acid receptors (FFAR2 and FFAR3), which are in the L enteroendocrine cells of the gut, and which produce the anorectic gut hormones, PYY and GLP-1. It has been suggested that these gastrointestinal hormones might be involved in the anorectic effects of fiber (Delzenne et al., 2005). Consistent with this idea both plasma PYY and GLP-1 were significantly higher in soluble fiber-fed rats compared to the cellulose-fed controls in the ***responders*** study. In the ***non-responders*** study, while PYY was also significantly higher in the soluble fiber-fed groups compared to the controls, for GLP-1 there was no clear-cut difference between the experimental and the control groups. Thus, both ***responders*** and ***non-responders*** showed elevated concentrations of PYY in the soluble dietary fiber-fed rat groups compared to the cellulose controls, yet food intake and body weight gain was reduced *only* in the ***responders*** study. This may suggest that decreased calorie intake requires the combined effects of PYY and GLP-1 (Neary et al., 2005), or that the satiety effects are more dependent on the action of GLP-1 than PYY (Gibbons et al., 2013). Alternatively, the anorectic effects of dietary fiber may be independent of the gut hormones PYY and GLP-1. There is support for this latter hypothesis, as some studies have reported that the release of gut satiety hormones is not dependent upon the activation of free fatty acid receptors by SCFAs (Lin et al., 2012; Christiansen et al., 2018) with others even suggesting that the actions of SCFAs on satiety and energy balance are independent of PYY or GLP-1 (Anastasovska et al., 2012; Frost et al., 2014). However, as the blood-gut hormone levels in this study were single-point (terminal) measurements, it is important not to over-interpret these single snapshot measurements.

An alternative mechanism could involve the direct effects of the SCFAs. For example, it has been suggested that acetate may provide the anorectic effects of dietary fiber through direct action on the hypothalamus, independently of gut hormones (Frost et al., 2014). In this study, acetate concentrations in the cecum were found to be significantly lower in the fiber-fed animals relative to the controls in the ***responder*** study (except pectin), yet higher in the fiber-fed animals than the controls in the ***non-responder*** study (except for the inulin group). Given the known difficulties in determining accurate levels of SCFA concentrations in the cecum, due to their rapid absorption, slow intraluminal diffusion rate, and metabolism by epithelial cells (Von Engelhardt et al., 1989; Takahashi, 2011; Nakatani et al., 2018; Sakata, 2019), the SCFA data from this study are open to question, and therefore neither refute nor support the idea of direct anorectic effects of acetate.

Given the conflicting evidence about the role of the SCFAs and satiety hormones in the control of energy balance in response to dietary fiber, this study further focused on the potential role of the gut microbiota, and specifically on correlations between the gut microbiota and some of the phenotypic changes in energy balance observed.

In both studies, the cecal microbiota of rats fed the cellulose control diets had a higher species richness (sobs) and estimated species richness (Chao) compared to the cecal microbiota from rats fed soluble dietary fibers. This suggests that the latter tend to promote bacteria that have the enzymatic capacity to degrade them or promote the abundance of bacteria that can utilize either the metabolic or substrate pathway of the products of primary degraders through cross-feeding (Rossi et al., 2005; Drew et al., 2018). As gut microbiota analysis based on sequence data provides an estimate of proportional rather than absolute representation, enhanced dominance of fiber-promoted groups will lead to a reciprocal reduction in the dominance of all other species, particularly those present in low proportional abundance, which will reduce the richness detected via sequencing. Additionally, the more diverse population observed in the cellulose control group in all the studies might be attributed to the poor fermentation of indigestible cellulose dietary fiber leading to the presence or adhesion of mucin bacteria in the cecal microbiota. Furthermore, the complex nature of cellulose can result in more diverse microbial communities than simple ones, as the complexity of the dietary fiber structure opens up more niches for microbial exploitation (Hamaker and Tuncil, 2014).

Analysis of beta diversity revealed a strong effect of diet on the microbiota composition of the different dietary groups in both studies. This is in line with other reports showing that different dietary fibers promote divergent bacterial communities, particularly in rodent models (Thomson et al., 2021). However, comparisons of the microbiota across the two studies revealed the cecal microbiota of rats given the same type of dietary fiber also clustered differently, which demonstrates that interindividual variation in the baseline gut microbiota composition is an important factor contributing to the response to dietary fiber (Murga-Garrido et al., 2021).

Given the stark differences in microbiota composition and weight loss between the two studies, we examined whether specific microbial taxa might be associated with weight change in rats. The results showed that the family *Erysipelotrichaceae* was significantly higher in proportional abundance in the cecal microbiota of rats in the soluble dietary fiber groups in the ***responders*** study where there was a significant decrease in body fat mass gain compared to the control cellulose group. An OTU classified as *Allobaculum fili*, a recently described species belonging to the family *Erysipelotrichaceae* (van Muijlwijk et al., 2023), was significantly higher in proportional abundance in the soluble dietary fiber groups compared to the control cellulose group in the ***responders*** study. It was completely absent in the ***non-responders*** study. Another study conducted by Meng et al. (2019) also demonstrated that, at the genus level of classification, the relative abundance of *Allobaculum* in the small intestine decreased correlatively with an increase in the body weight of rats. Although our moderate negative correlation was not as strong as those seen in the studies conducted by Ravussin et al. (2012) and Meng et al. (2019), it still points to a potential relationship between the *Allobaculum* genus, and related bacteria, with decreased fat mass in rodents.

In addition to the *Erysipelotrichaceae* findings, other microbiota features were potentially of interest. For example, the family *Enterobacteriaceae* from the phylum *Proteobacteria* was discovered to be proportionally more abundant in the cecal microbiota of rats whose diets were supplemented with 10% pectin in the ***responders study***. It has been reported that some members of the family *Enterobacteriaceae* have the enzymatic capability to degrade pectin, which might explain the abundance of this family thriving in the cecal microbiota of rats whose diet was supplemented with 10% pectin (Abbott and Boraston, 2008). It is also potentially noteworthy that the phylum *Proteobacteria* has been linked to perturbations in the gut microbiota (Shin et al., 2015). This could indicate that the weight loss observed in the initial ***responders*** study might have been due to a pathological impact of the elevated *Enterobacteriaceae* in the rats belonging to the pectin group. Further work would be required to test whether elevated *Enterobacteriaceae* levels alone cause weight loss in rats.

Potential limitations of the study are the different lengths of time and temperatures used for storage of the cecal samples prior to extraction and analysis, with greater loss of certain bacteria (e.g., *Bacteroidetes*) being likely with the longer times in storage (Bahl et al., 2012). The lower alpha diversity seen in the ***responders*** relative to the ***non-responders*** samples may be an indication of this. Nonetheless the most striking result in the study was the presence of *Allobaculum fili* in the ***responders*** (oldest samples) yet complete absence in the ***non-responders*** (newest samples), going against any prediction that differences in microbiota are simply related to time in storage. It is possible that we did not capture all the potential differences between the microbiotas from the two studies, due to differences in sample age and storage conditions. However, this would tend to underestimate rather than overestimate greater abundance in the ***responders*** samples, which may be related to the negative body weight. The fact that the data still point to significant differences between the microbiota of the rats between the two studies despite this suggests that the differences observed may underpin the differential the body weight and adiposity responses between the two studies.

Another factor that could have influenced the different outcomes of the studies is the nature and source of the fibers. While beta-glucan was sourced from the same supplier for both studies, this was not possible for pectin. It is known that during the processing of commercial-grade pectin, the majority of the neutral side chains are eliminated, thus, pectin can be divided into two main classes: high-esterified pectin that consists of a degree of esterification >50% (typically 55–75%), or low-esterified pectin which consists of a degree of esterification <50% (typically, 25–45%). Although sourced from different suppliers, the pectins used in both studies were pure apple pectin, both containing high methyl esterification of its galacturonic acid residues. Thus, the pectin used was as similar as possible between the two studies. Nonetheless, the fact that none of the fibers were effective in lowering body weight in the non-responders study, yet all were effective in the responders study suggests that it was not the type or nature of the fibers that was the limiting factor, rather it was something else, namely the microbiota.

While we did not analyze the background cecal microbiota before the commencement of our studies, the significant difference observed in the clustering (beta diversity) of the cecal microbiota of rats that were fed the same type of dietary fiber between the ***responders*** and ***non-responders*** study is a strong indication of how different the background cecal microbiota were at the start of the studies. It seems likely that these differences in baseline microbiota are the strongest determinant of the differential response. There are multiple factors that could have contributed to differences in gut microbiota at the start of the experiment. Firstly the animals were sourced from the same vendor (Charles River), but in markedly different years (2011 v 2017), behind which a variety of animal husbandry practices, including re-derivation, the use of antibiotics and drugs as well as differences in diet will have contributed to variation in gut microbiota (Ma et al., 2012; Franklin and Ericsson, 2017). Secondly, the experiments were conducted in different animal experimental facilities. This makes reproducibility in microbiota studies enormously challenging as it is difficult to control for all the potential environmental variations that could influence the gut microbiota.

## 5 Conclusion

This study provides evidence from rodent models showing that soluble fiber consumption can cause decreased body weight gain and fat mass, but the efficacy of this response may be related to the animals’ gut microbiota. Analysis of the composition of the cecal gut microbiota between rats in the ***responders*** and ***non-responders*** studies demonstrated a significant difference in microbiota response to dietary fiber feeding, even though the same type of dietary fiber was fed to the rats in each study. This indicates that inter-individual variation in the gut microbiota is a key factor in determining phenotypic response to dietary fiber interventions. Therefore, an important implication of these studies is that greater consideration needs to be given to the background gut microbiota profile of animals or humans when undertaking fiber intervention studies, as this is likely to affect the outcome response. The preponderance of *Allobaculum fili* in the cecal microbiota of rats in the soluble dietary fiber group of the ***responders*** study might suggest that some bacteria are more involved with a decrease in body weight and fat mass than others. Whether these bacteria are actively involved, or part of a bigger bacterial picture associated with decreased fat mass, requires further research.

## Data availability statement

The raw sequence reads (Fastq files) for this study were deposited to the National Center of Biotechnology Information (NCBI) Sequence Read Achieve (SRA) database under accession PRJNA1079537.

## Ethics statement

The animal study was approved by University of Aberdeen Animal Welfare and Ethics Review Board. The study was conducted in accordance with the local legislation and institutional requirements.

## Author contributions

SS: Conceptualization, Formal analysis, Investigation, Methodology, Visualization, Writing – original draft. AR: Conceptualization, Data curation, Funding acquisition, Investigation, Methodology, Project administration, Supervision, Writing – review & editing. AW: Supervision, Writing – review & editing. PM: Conceptualization, Funding acquisition, Investigation, Methodology, Project administration, Supervision, Writing – review & editing.

## Footnotes

^1^https://arriveguidelines.org/arrive-guidelines
