# Supplementary material for: Resident gut microbiota community determines the efficacy of soluble fiber in reducing adiposity
Source: Front Microbiol. 2024 Apr 30;15:1392016. doi: 10.3389/fmicb.2024.1392016 (PMC11091261; doi:10.3389/fmicb.2024.1392016)
Supplement: Supplementary file 1 [file Data_Sheet_1.zip › Supplementary Tables S1-S3.PDF]

Table S1 Composition of experimental diets for ***responders*** study
Re-used from (Adam *et al.*, 2014), under CC BY 3.0 licence.

**Diet**	**CONT**	**CELL**	**FOS**	**GLUC**	**PECT**
**Ingredients (% w/w)**					
Maize starch	46.57	41.57	41.57	41.57	41.57
Maltodextrin	15.5	15.5	15.5	15.5	15.5
Sucrose	10	10	10	10	10
Casein	14	14	14	14	14
Soyabean oil	4	4	4	4	4
AIN-93 Mineral mix	3.5	3.5	3.5	3.5	3.5
AIN-93 Vitamin mix	1	1	1	1	1
Choline bitartrate	0.25	0.25	0.25	0.25	0.25
L-cystine	0.18	0.18	0.18	0.18	0.18
Cellulose	5	10	0	0	0
FOS[2]	0	0	10	0	0
Beta-glucan[3]	0	0	0	10	0
Pectin[4]	0	0	0	0	10
ME[5] (MJ/kg)	15.7	15.2	15.2	15.2	15.2
GE[6] (MJ/kg)	17.2	17.4	17.2	17.1	17.1
**Composition (% w/w)**					
Dry matter	91.9	92.2	92.2	91.9	92.2
Total carbohydrate[7]	63.5	58.5	59.5	65.3	57.5
Total nitrogen[8]	2.0	2.0	2.0	2.1	2.1
Total fat[9]	4.5	5.0	5.2	4.7	4.7
NSP[10]- soluble	0.50	0.46	8.78[11]	7.74[12]	4.23
NSP[10]- insoluble	2.02	3.45	0.45	0.46	0.62

[1]All diets were based on AIN-93 M diet (American Society for Nutrition, Bethesda, MD USA) and manufactured by Special Diet Services Ltd. (Witham, Essex UK).

[2]Fructo-oligosaccharide (Higher Nature FOS Powder; Revital Ltd., Ruislip, Middlesex UK).

[3]Oat beta-glucan (Cambridge Commodities Ltd., Ely, Cambridgeshire UK).

[4]Apple pectin (Solgar Apple Pectin; Revital Ltd.).

[5]ME, metabolisable energy calculated from Atwater Fuel Energy of diet components.

[6]GE, gross energy determined using Gallenkamp Adiabatic Bomb Calorimeter.

[7]Total carbohydrate by standard acid-hydrolysis followed by colorimetric glucose determination.

[8]Total nitrogen determined using VarioMax CN Analyser.

[9]Total fat measured using standard acid-hydrolysis and gas chromatography.

[10]NSP, non-starch polysaccharides, analysed by the Englyst procedure [27], using gas chromatography and spectrophotometry to measure constituent sugars, and including [11]fructose detection for FOS diet by ultra-performance liquid chromatography.

[12]GLUC diet analysed specifically for beta-glucan by assay kit (K-BGLU; Megazyme, Bray, Co. Wicklow, Ireland).

Table S2 Composition of experimental diets ***non-responders*** study

**Diet**	**Control**	**INUL**	**GLUC**	**PECT**
**Ingredients (% w/w)**				
Maize Starch	46.57	41.57	41.57	41.57
Maltodextrin	15.5	15.5	15.5	15.5
Sucrose	10	10	10	10
Casein	14	14	14	14
Soyabean Oil	4	4	4	4
AIN93 Mineral mix	3.5	3.5	3.5	3.5
AIN93 Vitamin mix	1	1	1	1
Choline bitartrate	0.25	0.25	0.25	0.25
L-cystine	0.18	0.18	0.18	0.18
Cellulose	5	0	0	0
Inulin	0	10	0	0
Beta-glucan	0	0	10	0
Pectin	0	0	0	10

Table S3 Cumulative food intake and changes in body weight and composition in the ***responder*** study. Rats were given different types of dietary fibres for 4 weeks. Re-used from Adam *et al*., (2014), under CC BY 3.0 licence.

	**Diet group**[2]				
	**CONT**	**CELL**	**FOS**	**GLUC**	**PECT**
Cumulative food intake (g)	$543.5^{a} \pm 22.8$	$566.2^{a} \pm 16.1$	$448.6^{b} \pm 15.1$	$488.5^{b} \pm 16.5$	$442.5^{b} \pm 10.5$
Body weight gain (g)	$93.6^{a} \pm 8.23$	$87.0^{a} \pm 8.43$	$59.0^{b} \pm 5.39$	$59.3^{b} \pm 6.27$	$51.5^{b} \pm 6.77$
Body fat gain (g)	$20.6^{a} \pm 3.41$	$16.7^{a} \pm 3.81$	$-0.15^{b} \pm 2.07$	$2.25^{b} \pm 1.78$	$-9.31^{b} \pm 2.94$
Lean tissue gain (g)	59.0 ± 5.22	59.4 ± 4.17	49.8 ± 4.34	41.3 ± 8.06	55.8 ± 4.91
Initial total body fat (%)	8.22 ± 0.63	7.60 ± 0.76	7.81 ± 0.57	8.21 ± 0.59	8.71 ± 0.87
Initial total body lean (%)	80.1 ± 0.63	80.5 ± 0.72	80.1 ± 0.65	80.1 ± 0.60	79.2 ± 0.87
Final total body fat (%)	$10.4^{a} \pm 0.86$	$9.26^{a} \pm 1.03$	$6.81^{b} \pm 0.73$	$7.71^{b} \pm 0.58$	$6.08^{b} \pm 0.46$
Final total body lean (%)	$77.5^{a} \pm 0.93$	$78.7^{a} \pm 0.96$	$80.7^{b} \pm 0.81$	$79.1^{a,b} \pm 1.35$	$82.1^{b} \pm 0.43$

[1] Total body fat mass and lean tissue mass measured by MRI.

[2]Diets were control (CONT, 5% w/w cellulose) or contained 10% w/w fibre as cellulose (CELL), fructo-oligosaccharide (FOS), oat beta-glucan (GLUC) or apple pectin (PECT); n = 10/group. Values are mean ± s.e.m. Within rows, values without a common letter are significantly different ($P < 0.05$).
